# Supplementary material for: Impact of Menopausal Status and Recurrent UTIs on Symptoms, Severity, and Daily Life: Findings from an Online Survey of Women Reporting a Recent UTI
Source: Antibiotics (Basel). 2023 Jul 5;12(7):1150. doi: 10.3390/antibiotics12071150 (PMC10376736; doi:10.3390/antibiotics12071150)
Supplement: Supplementary file 1 [file antibiotics-12-01150-s001.zip › antibiotics-2458389-supplementary.pdf]

Supplementary material

Symptom reported	Menopausal status	N (%)	Adjusted OR (95% CI)
Nocturia	Pre-menopause	240 (42%)	Reference
	Menopause	175 (53%)	1.43 (1.05, 1.96)
	Post-menopause	100 (51%)	1.31 (0.84, 2.06)
Cloudy urine	Pre-menopause	197 (35%)	Reference
	Menopause	139 (42%)	1.27 (0.92, 1.74)
	Post-menopause	71 (36%)	1.03 (0.65, 1.63)
Dysuria	Pre-menopause	374 (66%)	Reference
	Menopause	229 (69%)	1.04 (0.74, 1.44)
	Post-menopause	111 (57%)	0.61 (0.38, 0.97)
Frequency	Pre-menopause	343 (60%)	Reference
	Menopause	236 (71%)	1.44 (1.03, 2.00)
	Post-menopause	136 (70%)	1.31 (0.80, 2.12)
Haematuria	Pre-menopause	85 (15%)	Reference
	Menopause	37 (11%)	0.58 (0.36, 0.94)
	Post-menopause	19 (10%)	0.46 (0.23, 0.93)
Incontinence	Pre-menopause	60 (11%)	Reference
	Menopause	52 (16%)	1.66 (1.06, 2.62)
	Post-menopause	45 (23%)	2.76 (1.50, 5.09)
Pain in lower abdomen	Pre-menopause	301 (53%)	Reference
	Menopause	165 (50%)	0.78 (0.57, 1.06)
	Post-menopause	83 (43%)	0.56 (0.35, 0.87)
Vaginal discharge	Pre-menopause	158 (28%)	Reference
	Menopause	32 (10%)	0.25 (0.16, 0.40)
	Post-menopause	13 (7%)	0.15 (0.07, 0.31)
Increased confusion	Pre-menopause	40 (7%)	Reference
	Menopause	15 (5%)	0.81 (0.42, 1.59)
	Post-menopause	8 (4%)	1.27 (0.45, 3.53)
Unsteady on feet	Pre-menopause	59 (10%)	Reference
	Menopause	21 (6%)	0.55 (0.31, 0.99)
	Post-menopause	15 (8%)	0.71 (0.31, 1.60)
Lower temperature	Pre-menopause	36 (6%)	Reference
	Menopause	6 (2%)	0.46 (0.19, 1.16)
	Post-menopause	3 (2%)	0.74 (0.17, 3.11)
Fever	Pre-menopause	98 (17%)	Reference
	Menopause	40 (12%)	0.69 (0.44, 1.08)
	Post-menopause	8 (4%)	0.25 (0.11, 0.60)
Rigors/shivering/shaking	Pre-menopause	67 (12%)	Reference
	Menopause	27 (8%)	0.66 (0.39, 1.21)
	Post-menopause	15 (8%)	0.66 (0.30, 1.42)
Kidney pain	Pre-menopause	156 (27%)	Reference
	Menopause	84 (25%)	0.88 (0.62, 1.26)
	Post-menopause	39 (20%)	0.58 (0.34, 0.99)
Strongly predictive symptoms *Two or more of nocturia, cloudy urine, dysuria*	Pre-menopause	466 (82%%)	Reference
	Menopause	300 (90%)	1.28 (0.93, 1.75)
	Post-menopause	166 (86%)	0.82 (0.52, 1.29)

Supplementary Table S1. Prevalence of self-reported symptoms and odds of having the symptoms in the menopausal (45-64 years, n=332) or post-menopausal (>65 years, n=194) group compared to the pre-menopausal (16-44 years, n=570) group. Percentages are the proportion of women within each menopausal group describing each symptom with their most recent UTI.

Symptom reported	UTIs in the previous year	N (%)	Adjusted OR (95% CI)
Nocturia	1-2 UTIs	440 (47%)	Reference
	3 or more UTIs	75 (46%)	0.94 (0.67, 1.33)
Cloudy urine	1-2 UTIs	335 (36%)	Reference
	3 or more UTIs	71 (43%)	1.43 (1.01, 2.03)
Dysuria	1-2 UTIs	622 (67%)	Reference
	3 or more UTIs	91 (55%)	0.63 (0.44, 0.89)
Frequency	1-2 UTIs	614 (66%)	Reference
	3 or more UTIs	101 (62%)	0.90 (0.63, 1.29)
Haematuria	1-2 UTIs	121 (13%)	Reference
	3 or more UTIs	19 (12%)	0.90 (0.53, 1.53)
Incontinence	1-2 UTIs	125 (13%)	Reference
	3 or more UTIs	32 (20%)	1.47 (0.93, 2.31)
Pain in lower abdomen	1-2 UTIs	459 (49%)	Reference
	3 or more UTIs	90 (55%)	1.27 (0.90, 1.79)
Vaginal discharge	1-2 UTIs	166 (18%)	Reference
	3 or more UTIs	37 (23%)	1.29 (0.82, 1.96)
Increased confusion	1-2 UTIs	50 (5%)	Reference
	3 or more UTIs	13 (8%)	1.44 (0.74, 2.81)
Unsteady on feet	1-2 UTIs	71 (8%)	Reference
	3 or more UTIs	24 (14%)	1.85 (1.10, 3.10)
Lower temperature	1-2 UTIs	37 (4%)	Reference
	3 or more UTIs	9 (5%)	1.23 (0.55, 2.76)
Fever	1-2 UTIs	118 (13%)	Reference
	3 or more UTIs	27 (17%)	1.46 (0.91, 2.35)
Rigors/shivering/shaking	1-2 UTIs	79 (9%)	Reference
	3 or more UTIs	29 (18%)	2.30 (1.42, 3.73)
Kidney pain	1-2 UTIs	222 (24%)	Reference
	3 or more UTIs	56 (34%)	1.71 (1.18, 2.47)
Strongly predictive symptoms<br>*Two or more of nocturia, cloudy urine, dysuria*	1-2 UTIs	802 (86%)	Reference
	3 or more UTIs	130 (79%)	1.03 (0.73, 1.46)

Supplementary Table S2. Prevalence of self-reported symptoms of an acute UTI and odds of that symptoms in women with recurrent UTI (3 or more UTIs in the previous year, n=164) compared to those with-out recurrent UTIs (1-2 UTIs in the previous year, n=931).

		Severity score N (%)											
		0	1	2	3	4	5	6	7	8	9	10	Total
Time from last UTI	< 3 months	7 (2%)	0 (0%)	6 (2%)	19 (6%)	20 (6%)	53 (16%)	55 (16%)	68 (20%)	60 (18%)	23 (7%)	23 (7%)	335
	3-6 months	3 (1%)	3 (1%)	8 (3%)	17 (5%)	25 (8%)	45 (14%)	70 (22%)	72(23%)	45 (14%)	23 (7%)	6 (2%)	318
	6-9 months	0 (0%)	2 (1%)	6 (3%)	14 (7%)	16 (8%)	25 (12%)	45 (22%)	56 (27%)	31 (15%)	11 (5%)	2 (1%)	207
	9-12 months	1 (0%)	9 (5%)	10 (5%)	21 (10%)	16 (8%)	33 (16%)	37 (18%)	38 (18%)	24 (12%)	6 (3%)	12 (6%)	206

Supplementary Table S3. Severity score according to time from last UTI. Note that 25 participants did not know how severe their last UTI was.
